# On the Cryptic Speciation in the Mosses with East Asia–East North America Disjunction: A Case Study of Two Poorly Understood Mosses from the Southern Extremity of the Russian Far East

**DOI:** 10.3390/plants13243558

**Published:** 2024-12-20

**Authors:** Vladimir E. Fedosov, Olga Yu. Pisarenko, Alina V. Fedorova, Olga M. Afonina, Elena A. Ignatova

**Affiliations:** 1 Geobotany Department, Biological Faculty, Lomonosov Moscow State University, 119234 Moscow, Russia; arctoa@list.ru; 2 Central Siberian Botanical Garden, Zolotodolinskaya 101, 630090 Novosibirsk, Russia; o_pisarenko@mail.ru; 3 Tsitsin Main Botanical Garden, Russian Academy of Sciences, Botanicheskaya 4, 127276 Moscow, Russia; alina_77777@mail.ru; 4 Komarov Botanical Institute, Russian Academy of Sciences, Prof. Popov Str., 2, 197022 St. Petersburg, Russia; stereodon@yandex.ru

**Keywords:** biogeography, biodiversity, DNA barcoding, arcto-tertiary flora

## Abstract

A survey of the moss flora of the southernmost part of the Russian Primorsky Territory yielded several intriguing taxa, whose identity is assessed herein based on an integrative morpho-molecular approach. *Bellibarbula recurva* was previously known in inland Asia only from the Sino-Himalayan region and the new locality is distant from the earlier known ones to ca. 3000 km. Despite the morphological uniformity, Russian specimens are remarkably distinct in sequences of all three obtained DNA markers, approaching an American specimen in the *rps4* sequence. Another probable relic, *Symblepharis* cf. *crispifolia*, appeared to be fairly common in the southern part of the Primorsky Territory, where low mountains are covered with hard-leaved forests. Russian specimens of *Symblepharis* cf. *crispifolia* var. *brevipes* show significant divergence from *S. crispifolia* s.str., which also has complex phylogenetic structure, obscuring further taxonomic implications. The description and illustrations of both taxa based on Russian specimens are provided, and the area, where both species occur, is briefly characterized; it includes numerous thermophilous species, which are rare or do not occur northwards. Our case study uncovers the problem of cryptic speciation within species distributed in temperate climate and is considered to represent relics of Arcto-Tertiary flora.

## 1. Introduction

Recent studies of biodiversity of mosses in the southern part of the Primorsky Territory, constituting the southernmost area of the Russian Far East, brought numerous records of previously undescribed or unknown Russian species [1,2,3,4,5,6], etc. Moreover, morpho-molecular revisions of several groups revealed many earlier neglected and misunderstood taxa occurring there [7,8,9,10,11]. However, most field trips were conducted in the southern spurs of Sikhote-Alin Mountains, which were considered especially rich in terms of the number of moss taxa, while broad-leaved forest and forest-steppe biomes situated southwards remain insufficiently studied [12,13]. A recent survey by [14] contributed to our knowledge of bryophyte diversity of the temperate broad-leaved forests of the Primorsky Territory, but its southernmost part still remains insufficiently covered by bryophyte collections, especially recent ones, which could be checked for their identity with molecular markers. At the same time, an assessment of moss diversity in this area is critical for the preparation of the handbook of the moss flora of Russia since many southern taxa considered as relics of Arcto-Tertiary flora (considered here according to [15]) are known in Russia only from here [16,17,18], and the potential number of taxa representing this phytogeographical element in the moss flora of Russia may increase. On the other hand, moss flora of NE China also remains poorly studied as it was exemplified by the aforementioned discoveries.

Therefore, in September 2024, V.F. and O.P. undertook a brief survey of bryophyte diversity in the southern extremity of the Primorsky Territory southwards to Reyd Pallada Bay of the Sea of Japan. These mountains represent the remnants of an Early Jurassic paleovolcano. In the modern relief, the preserved raised blocks of the previously single volcanic structure are expressed as steep rocky outcrops; the largest of them (the Mramornaya and Priozernaya Mts.) rise to 280 m above sea level with a length of several kilometers. They are composed of rich-in-magnesium basalts, calc-alcaline andesites, dacites, and rhyolites [19]. The area of the southern extremity of the Primorsky Territory is distinguished by specific climatic conditions [16,20]. The Black Mountain ridge protects the area from the northern and northwestern cold winter winds, allowing warm air from the south and southeast to flow in, and the East-Korean Current warms the area in summer. Therefore, the largest sum of positive temperatures for the Primorsky Territory (>3000 °C) was registered here; the average annual air temperature is also highest in the Primorsky Territory, 1-2 degrees higher than in Vladivostok (up to +7.2 °C in Khasan), while the average annual soil surface temperature is 3 degrees higher than in Vladivostok (up to +8 °C). The frost-free period is also the longest in the region, 194 days on average. The proximity to the sea increases the amount of precipitation (675–760 mm) and fog exposure. Already, L.V. Komarov mentioned that many plant species can be found in Russia only in the “Posyet area” [21] and further studies confirmed high specificity of flora of the area [18].

**Figure 1:**
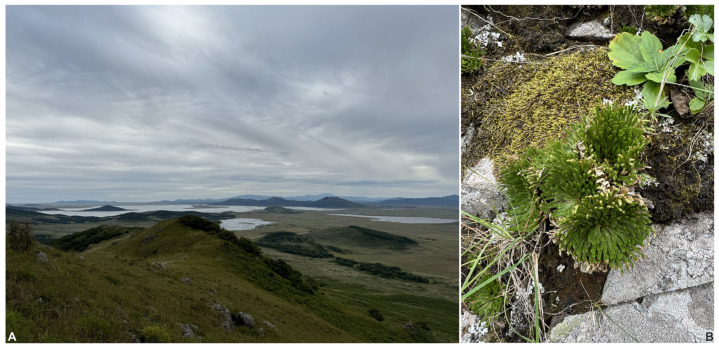
Habitat of *Bellibarbula* cf. *recurva* and *Symblepharis crispifolia* in southern extremity of Russian Far East. (**A**) Mramornaya Mt., view from summit; (**B**) rock outcrops with *Lycopodioides tamariscina* and *Hypnum leptothallum* and spots of bare soil—characteristic ecotope where *Bellibarbula* cf. *recurva* occurs.

On the slopes of the surveyed mountains, the largest areas are dominated by communities of tall grasses and herbs (*Miscanthus purpurascens* Anderss, *Arundinella anomala* Steud., *Calamagrostis langsdorffii* (Link) Trin., *Artemisia umbrosa* (Bess.) Turcz. ex DC., *A. mandshutica* (Kom.) Kom., *Ptarmica ptarmicoides* (Maxim.) Worosch., and others) with a high abundance of shrubs (*Corylus heterophylla* Fisch. ex Trautv., *Lespedeza bicolor* Turcz., *L. cyrtobotrya* Miq., *Rhododendron schloppenbachii* Maxim), intermingled with crooked *Quercus mongolica* Fisch. ex Ledeb. and *Q. dentata* Thunb. groves and rock outcrops (Figure 1). *Lycopodioides tamariscina* (P. Beauv.) Tzvel. is abundant on rocks together with bryophytes. Among the latter, several thermophilous species like, e.g., *Campylopus subulatus* Schimp. ex Milde, *Glyphomitrium ambiguum* Fedosov, Ignatova & Ignatov, and *Chionoloma cylindrotheca* (Mitt.) M. Alonso, M.J. Cano & J.A. Jiménez, occur, although, in general, diversity of mosses is not very high.

Several peculiar specimens of saxicolous pottioids were collected from rock outcrops of Mramornaya and Priozernaya Mountains; they combined a *Bryoerythrophyllum*-like appearance, numerous small C-shaped papillae per cell, and weakly differentiated short-rectangular basal leaf cells. These specimens attracted our attention due to their possible assignment in the genus *Bellibarbula* P.C. Chen, which has previously been reported in Asia only from the Sino-Himalayan region. Alternatively, these plants could represent an insufficiently known taxon of *Bryoerythrophyllum,* the diversity of which remains weakly understood in East Asia, as was shown by, e.g., Kučera *et al.* [22]. Among other records of phytogeographically interesting and insufficiently understood bryophytes, *Symblepharis* cf. *crispifolia* (Mitt.) Fedosov, M. Stech & Ignatov represents a remarkable example of distribution, largely associated with mixed and deciduous forests of the Primorsky Territory. This taxon, previously known from Russia based on a few old records, was not included in the molecular phylogenetic study by Fedosov et al. [23] and its classification in the genus *Symblepharis* is based largely on morphology and topology of splitstree, published by Hedenäs [24]. However, it was recently found in many localities in the southern part of the Primorsky Territory; moreover, due to having rather short setae and emergent capsules, all collected specimens corresponded to “*Oncophorus crispifolius* var. *brevipes*”, which has never been a matter of taxonomic reassessment with the morpho-molecular approach. Therefore, the identity of these taxa assessed through the integrative approach is considered in the present study.

## 2. Results

### 2.1. Molecular Assessment of Phylogenetic Affinities

**Figure 2:**
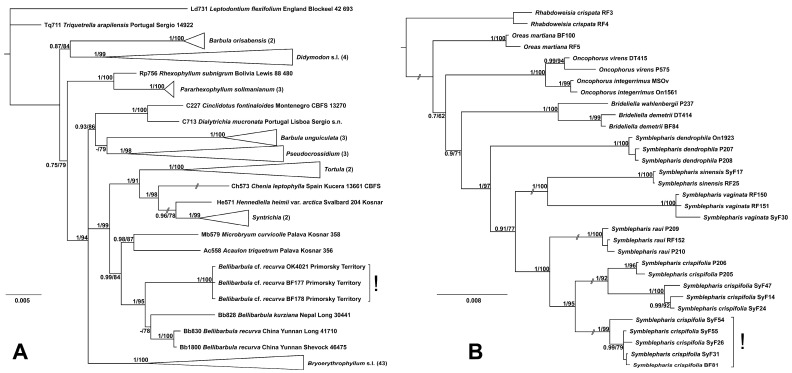
Bayesian phylogenetic trees, showing affinities of target groups of specimens (marked with “!”). (**A**): *Bellibarbula* cf. *recurva* inferred from the combined plastid *rps4*-*trnS* and *trnM*-*trnV* dataset; (**B**) *Symblepharis crispifolia* var. *brevipes* inferred from the combined cp *trnF*-*trnT*, *rps4*-*trnS*, *trnG*, mt *Nad5*, and nr ITS dataset. Bayesian posterior probabilities inferred from the datasets with indels coded using a simple indel coding approach, and Bootstrap values obtained from 1000 pseudoreplicates of ultrafast bootstrapping are shown above the branches.

Topologies of the trees inferred from combined datasets were identical for ML and Bayesian inference with coded indels. In the tree focused on the resolving affinities of the *Bellibarbula*-like plants (Figure 2A), three tested specimens formed a completely supported clade within the highly supported *Bellibarbula* clade (PP = 1; BS = 95). This clade occupied the position sister to the not supported clade, which captures the remaining accessions of *Bellibarbula* included in the analysis, representing Sino-Himalayan specimens of both *B. recurva* and *B. kurziana*. Such topology suggests referring the plants from the Primorsky Territory of the Russian Far East to the new species; however, morphologically, it corresponds well to the circumscription of *B. recurva* (see Discussion). Therefore, we report *B. recurva* as a new species for Russian moss flora and provide its description based on Russian specimens and comments on its ecology.

All four Russian specimens of *Symblepharis* cf. *crispifolia* form a highly statistically supported clade (PP = 1; BS = 99), where one Japanese accession was found (Figure 2B). This clade is sister to a moderately supported clade (PP = 1; BS = 99) composed of Japanese and Korean accessions, within which two clades correspond to the originally studied specimens and those studied by Hedenäs (2017). The clade corresponding to “*S. crispifolia* s.l.” (PP = 1; BS = 95) forms a completely supported grouping with the *S. raui* clade. Specimens in the clade, which accommodate Russian accessions of *S. crispifolia* var. *brevipes*, have somewhat different sequences; along with minor variation in sequences among them, the specimen SyF54 from Priozernaya Mt. (the area where *Bellibarbula* was found) does not have several synapomorphies, shared by the remaining four accessions, including the Japanese one. Below, we provide a description of the taxon based exclusively on the plants from the clade, which accommodates specimens from the Russian Far East.

### 2.2. Morphology

**Figure 3:**
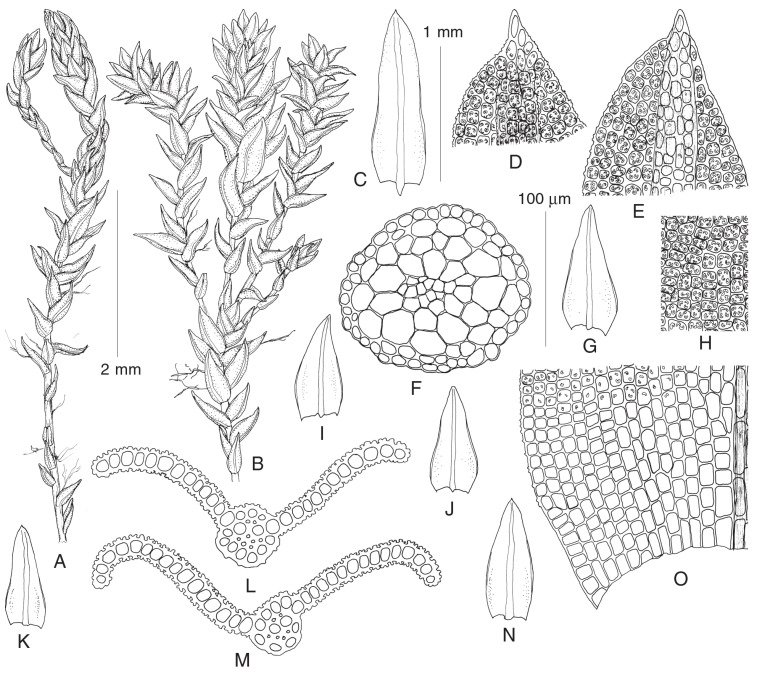
Line drawings of gametophyte of *Bellibarbula recurva* (from: Russia, Primorsky Territory, *Fedosov & Pisarenko* MW9133374): (**A**) habit, dry; (**B**) habit, wet; (**C**,**G**,**I**–**K**,**N**) stem leaves; (**D**,**E**) upper leaf cells; (**F**) stem transverse section; (**H**) median laminal cells; (**L**,**M**) leaf transverse sections; (**O**) basal leaf cells. Scale bars: 2 mm for (**A**,**B**); 1 mm for (**C**,**G**,**I**–**K**,**N**); 100 µm for (**D**–**F**,**H**,**L**,**M**,**O**).

***Bellibarbula recurva*** (Griff.) R.H. Zander, Bull. Buffalo Soc. Nat. Sci. 32: 142. 1993.—*Gymnostomum recurvum* Griff., Calcutta J. Nat. Hist. 2: 482. 1842.—*Bryoerythrophyllum recurvum* (Griff.) K. Saito, Fl. E. Himalaya 3: 254. 1975. (Figure 3).

Plants small- to medium-sized, in loose or dense turfs, green, olive, brownish or reddish-brown. Stem: 0.3–0.7 (–1) cm, with central strand, erect, forked, evenly foliate. Leaves appressed when dry, sometimes with the tips of the longest leaves bent to the side, spreading when moist, 0.6–0.8 (–1.2) × 0.25–0.35 (–0.4) ovate to ovate-lanceolate, acute or subobtuse due to eroded apical cells, usually with one or several smooth brownish cells at apex. Margins entire, recurved in mid-leaf, unistratose. Costa single, strong, occupying 1/10–1/6 of leaf base width, percurrent, slightly wavy distally or straight, smooth on the dorsal surface, covered by short chlorophyllous papillose cells on ventral surface, in transverse section biconvex, with differentiated dorsal and ventral epidermis, one row of guide cells, one layer of ventral stereids and two layers of dorsal stereids. Leaf lamina unistratose; upper laminal cells 10–13 × 7–10 µm, rounded-quadrate and short-rectangular, thin-walled, with 4–8 per cell or more, tiny, C-shaped papillae; basal laminal cells weakly delimited from upper cells, short-rectangular, 9–20 (–25) × 8–11 µm, in few rows along insertion smooth. Gametangia and sporophytes not seen in plants from Russia.

Plants in four specimens from the relatively compact area show a considerable variation in plant size, color, and leaf surface ornamentation: in exposed sites where scattered plants of *B. recurva* occur among liverworts, they are small, hardly reaching 2–3 mm in height, and red-brownish, with leaves up to 0.8 mm and with comparatively massive papillae, while in more sheltered sites, plants form dense, green to olivaceous tufts up to 1 cm high, leaves reach 1.2 mm in length, and papillae on leaf surfaces are more slender.

Specimens examined:

Russia: Primorsky Territory, Khasansky Distr., vicinity of Khasan Settl., Reid Pallady Bay southern shore, Mramornaja Mt. NW slope near ridge (42.56695N, 130.80278E, 183 m. alt.) meadow with oaks (*Quercus mongolica, Q. dentata*), bushes, and rock outcrops, on cliff ledges along with *Hypnum leptothallum*, 21.IX.2024, *V.E. Fedosov & O.Yu. Pisarenko* (MW9133374 isolate BF177 ITS: PQ590637, *rps4*-*trnS*: PQ593607, *trnMV*: PQ593624; MW9133376 isolate BF178 ITS: PQ590638, *rps4*-*trnS*: PQ593608, *trnMV*: PQ593625; MW9133394, MW9133397); the same area, Priozernaya Mt. NW slope (42.54342N, 130.70530E, 64 m alt.) meadow with rock outcrops, in niche, 22.IX.2024, *V.E. Fedosov & O.Yu. Pisarenko* (MW9133391 isolate OK4021 ITS: PQ590636, *rps4*-*trnS*: PQ593609, *trnMV*: PQ593623).

**Figure 4:**
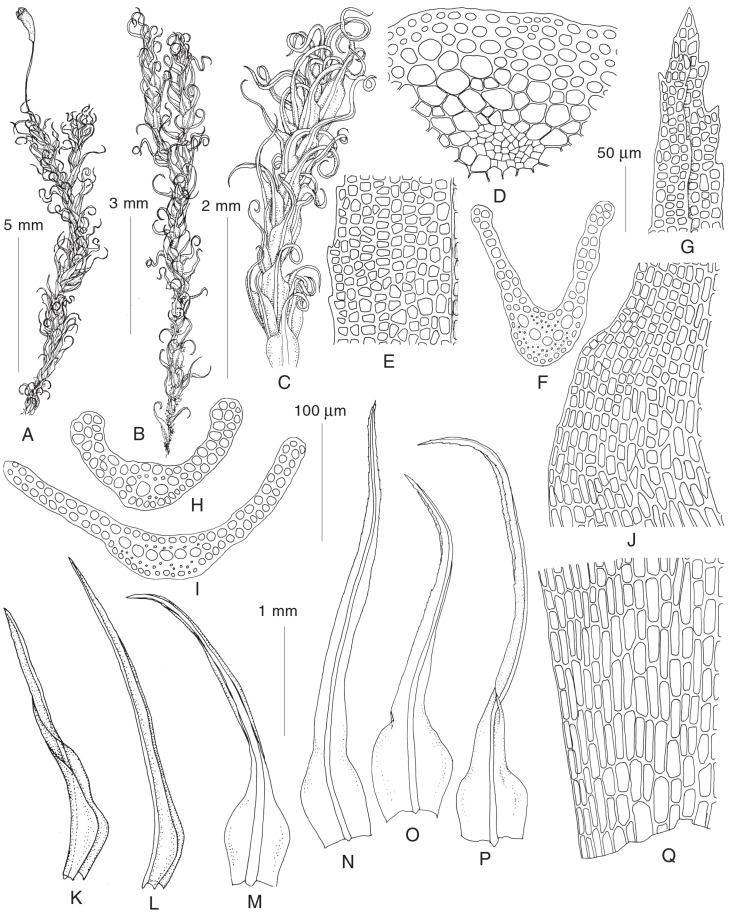
Line drawings of sporophyte and gametophyte of *Symblepharis crispifolia* s.str. (**A**,**E**–**G**,**J**,**N**–**Q** from Russia, Primorsky Territory, *Lazarenko* 20.X.1933) and “*S. crispifolia* var. *brevipes*” (**B**–**D**,**H**,**I**,**K**–**M** from Russia, Primorsky Territory, Lazovsky Reserve, 24.IX.1987 *Bardunov et al.*, VLA): (**A**–**C**) habit, dry; (**D**) stem transverse section; (**G**) upper leaf cells; (**E**) median laminal cells; (**F**,**K**–**P**) leaf transverse sections; (**J**) cells of leaf shoulder; (**K**–**P**) stem leaves; (**Q**) basal leaf cells. Scale bars: 5 mm for (**A**); 3 mm for (**B**); 2 mm for (**C**); 1 mm and 200 µm for (**K**–**P**); 100 µm for (**F**,**H**,**I**); 50 µm for (**D**–**J**,**Q**).

***Symblepharis crispifolia*** (Mitt.) Fedosov, M. Stech & Ignatov, Bot. J. Linn. Soc. 195(4): 562. 2021 [2020].—*Didymodon crispifolius* Mitt., J. Linn. Soc., Bot. 8: 148. 1865 [1864].—*Oncophorus crispifolius* (Mitt.) Lindb., Contr. Fl. Crypt. As. 229. 1872 [1873]. (Figure 4; for color images, see [14]).

Plants medium-sized, in loose tufts, dark green. Stem up to 1.5 cm long. Leaves strongly crisped when dry, with scarcely appressed bases and spreading acumina when wet, 2.0–2.8 × 0.3–0.5 mm, from ovate base abruptly narrowed into a long, linear-lanceolate to linear, canaliculate acumen. Margins plane, serrate, bistratose in 3–4 cell rows. Costa strong, occupying 1/7–1/5 of leaf base width, ending few cells below leaf apex, smooth on the dorsal side, with differentiated dorsal and ventral epidermis, one row of guide cells and two stereid bands. Leaf lamina unistratose proximally, bistratose with unistratose patches, completely bistratose or with tristratose patches distally; upper laminal cells ca. 6–15 × 8 µm, rounded-quadrate, short-rectangular and transverse-rectangular, smooth or weakly mammillose, thin- to moderately thick-walled; basal laminal cells, 30–60 × 10–15 µm, short-rectangular, with moderately thickened, not sinuose and not porose walls, yellowish. Setae 3–5 (−10) mm, yellow or brownish, straight to curved. Capsules 1.2–1.4 mm long, oblong, asymmetric, curved, longitudinally furrowed when dry, strongly or rather weakly strumose, brownish. Opercula with long oblique beak. Annulus not differentiated. Peristome dicranoid, teeth ca. 400 µm long, unequally split to the half of their length, longitudinally striolate below, papillose above. Spores, 16–20 µm. Calyptrae cucullate.

Most specimens from the Russian Far East are rather uniform in plant size, leaf shape, setae length, etc., but show several variability in coloration (mostly agreeing with exposure), upper leaf cell areolation (smooth to rather distinctly mammillose), and capsule shape (many specimens have weaker strumose capsules). Most specimens from the “*S*. *crispifolia* s.str.” clade have stronger dentate upper leaf margins and only partly bistratose upper leaf lamina, while the specimens of “var. *brevipes*” typically have weak and blunt teeth in the upper leaf portion and completely bistratose or partly tristratose upper leaf lamina. On the other hand, all specimens from the “*S*. *crispifolia* s.str.” clade (SyF47, 14, 24) possess longer setae (mostly 0.7–1 cm); otherwise, they are rather variable in leaf shape, possessing both abruptly and gradually narrowed leaves. However, the specimen SyF26 from Japan, which morphologically corresponds to the clade where other Japanese and Korean specimens were found, appeared nearly identical to the specimens of “var. *brevipes*” from the Primorsky Territory based on molecular data. Finally, the plants from Hualaza Mt. in the south extremity of the Sikhote-Alin Mountains had setae longer than 0.5 mm and strongly dentate upper leaf lamina as it is characteristic to *S. crispifolia* s.str. (Figure 4), but the specimen is too old to be checked with the molecular data. Although this species was considered rare in the Primorsky Territory and until recently most reports dated to 30–70 years of the 20th century, our recent collections show that this is a common saxicolous moss in the low mountain relief of the southern part of the Primorsky Territory, covered by oak-dominated and polydominant broad-leaved deciduous forests.

Specimens examined:

Russia, Primorsky Territory, Lazovsky Distr., Lazovsky State Reserve, Perekatnaya River middle course, N-faced rock outcrops, 29.IX.1974, *L.V. Bardunov, A. Olinovich & O. Kirchakova* (LE); the same area and place, on rocks, 24.IX. 1987, *L.V. Bardunov, V.Ya. Cherdantseva & Druzhinina* (VLA); Partizansk Distr., vicinity of Lozovyj Settl., upper course of spring, N-faced rock outcrops, 13.IX.1974, *L.V. Bardunov, V.Ya. Cherdantseva & A. Olinovich* (LE); Muravjova-Amurskogo Peninsula, Sovetsky Distr., vicinity of Shamara Bay, on rocks, 26.VIII.1930, *A.S. Lazarenko* (LE); Vladivostok city, vicinity of Okeanskaya station, Koreyskaya Mt., on cliffs, 10.VIII.1930, *A.S. Lazarenko* (LE); the same area, N-faced slope of hill (43.21376 N, 131.97727 E, 212 m alt.), polydominant forest with *Abies holophylla*, on rocks and linden (*Tilia amurensis* Rupr.), 15.IX.2024, *V.E. Fedosov & O.Yu. Pisarenko* (MW9133381*, MW9133382, MW9133384); Shkotovsky Distr., Hualaza Mt., ca. 600 m alt., cliffs above the Smol’ny Klyuch Creek, 20.X.1933, *A.S. Lazarenko* (LE); Ussurijsky State Reserve, vicinity of Peyshula Field Station, S-faced slope of Zmeinaya Mt., 20.VIII.2022, *V.E. Fedosov, Yu.S. Ishchenko & A.V. Shkurko* (MW9130807, MW9132776*); Khasansky Distr., vicinity of Barabash settl., Zemlya Leoparda National Park, ecological path (43.18036N, 131.48921E, ca. 12 m. alt.), polydominant broad-leaved forest, on shaded boulder, 29.IX.2023, *V.E. Fedosov* (MW9133379*); Andreevka settl. vicinity (42.61924N, 131.17718E, 295 m alt.), path along the ridge, meadow with rock outcrops and separate trees, on rocks, 19.IX.2024, *V.E. Fedosov & O.Yu. Pisarenko* (MW9133378); the same area, forested slope of hill (42.66316N, 131.15459E, 177 m alt.), dry oak-dominated forest with rock outcrops, on shaded boulder, 18.IX.2024, *V.E. Fedosov* (MW9133377); Khasan Settl. vicinity, Reid Pallady Bay southern shore, Mramornaja Mt. NW slope near ridge (42.56695N, 130.80278E, 175–210 m alt.), meadow with oaks, bushes, and rock outcrops, on dry shaded vertical surface of cliff, 21.IX.2024, *V.E. Fedosov & O.Yu. Pisarenko* (MW9133395, MW9133398); the same area, Priozernaya Mt. NW slope (42.54342N, 130.70530E, 64 m alt.), meadow with rock outcrops, on overhanging surface of boulder, 22.IX.2024, *V.E. Fedosov & O.Yu. Pisarenko* (MW1933392*).

South Korea: Cheju Island, Hallasan National Park, Sogwipo-shi, Bopchong-dong Distr., Yongsil Trail Course, *Pinus densifolia* and *Quercus mongolica* forest, 1000–1600 m alt., on rocks, 13.VII.2000, *Si He & Jong-Suk Song 34,639* (MW9117607); Tongduch’on-shi co, Camp Casey, November Unit, along eastern ridge of Soyasan, about 2 km east of Tongduch’on, 400 m. alt, mixed hardwood forest, on gneiss rocks, 11.VIII.1997, *J.R. Shevock 16,189* (MW9117624); South Gyeongsang Province, Geoje-city, Dundeok-myeon, Eoongol stream, 360 m alt., deciduous forest in stream valley, on cliff, 6.XII.2014, *V.A. Bakalin K-43-16-14* (VGBI); Gyeonggi-do, Goyang-si, Deokyang-gu, Bukhan-dong, Buchansan National Park (37.65389 N, 126.99388 E, 157 m. alt.), 25.V.2024, S.S. Choi 240131*.

Japan: Honshu, Ibaraki Pref., Mt. Tsukuba, Tsukuba Shrine, on shaded stone wall in *Cryptomeria japonica* forest, 250 m alt., 17.V.2000, *M. Higuchi* (Bryophyta Selecta Exciccata 1162); Hiroshima Pref., Kure-shi, Mt. Noro, 800 m alt, mixed forest on ridge, cliff in partial shade, 9.X.2014, *V.A. Bakalin J-41-13-14* (VGBI); Kyushu, Kagoshima-ken, Kumage-gun, Yakushima-cho, ca. 540 m alt., on boulder, 4.III.2014 *Y. Sakamoto* (Bryophytes of Asia 509, MW9050532*); Miyazaki Prefecture, Inohae Valley north of Nichinan, evergreen south-temperate forest in deep valley, on cliff, 9.XII.1998, *M.S. Ignatov & E.A. Ignatova 98–529* (MW9050531); Fukuoka Pref., Tagama-gun, Soeda-machi, Hiko-san Mt., along upper course of Shioi River, 770–900 alt., mixed forest along stream, boulder in partial shade, III.2014, *V.A. Bakalin J-7-23-14* (VGBI).

Note: An asterisk indicates specimens involved in the molecular study.

## 3. Discussion

### 3.1. Molecular Affinities

Morphological specificity of the genus *Bellibarbula* includes basal leaf cells scarcely differentiated from those in the middle and upper parts of leaf lamina, and convolute, sheathing perichaetial leaves. These characters differentiate its species from the superficially similar short-leaved species of the genus *Bryoerythrophyllum* [25,26]. *Bellibarbula* currently includes two species, *B. kurziana* P.C. Chen, an endemic of the Sino-Himalayan region, and widely distributed *B. recurva*, which was transferred into this genus by R.H. Zander [27]; the placement of the latter species was later supported by Blockeel et al. [28] and Kučera et al. [22]. *Bellibarbula recurva* was described from the Indian Himalaya and since then has also been reported from Nepal, Bhutan, China (Yunnan, Tibet), Africa, New Guinea, a few places in the Appalachian region of the USA, and Central and South America [26,29,30]. In Asia, most localities of this species are concentrated in the Sino-Himalayan region; so, the new record from the coastal area of the Sea of Japan is distant at ca. 3000 km from the closest known localities. Although plants collected in Russia lack perichaetia, a weak differentiation of lower leaf cells aligns well with the circumscription of the genus; these specimens completely agree with the description and illustrations, provided by Li et al. [31] for *B. recurva*, disagreeing with *B. kurziana* due to acute pointed vs. obtuse leaf tips. Similarly to plants from the USA described by [26], plants from the Primorsky Territory sometimes have costae slightly sinuose distally. Moreover, the sequence of the *rps4*-*trnS* region of the plastid genome available in GenBank for an American specimen (AY908032, United States, South Carolina, Oconee Co., Lower Bear Camp Creek gorge, Lake Jocassee, *Anderson 24,981* (DUKE)) shows seven synapomorphies with our sequences, not shared by specimens from the Sino-Himalaya, and three outapomorphies shared neither by our specimens nor by those from the Sino-Himalaya. Closer relation between plants from the Primorsky Territory and the specimen from South Carolina may indicate a rather recent origin of disjunction between East Asian and North American parts of the species distribution, which otherwise can be considered as typical for Arcto-Tertiary relicts [32,33]. The possible wider distribution of the species in the tropics may align it with the widespread-in-low-altitude and highly polymorphic *Hyophila*, which also appeared as problematic in terms of taxonomy [27,34]. The deeper study of Sino-Himalayan specimens including the type of *Gymnostomum recurvum* and types of putative synonyms of *B. recurva* (see [27,35,36,37,38]) and also specimens from the other regions of the species distribution (Africa, North and Central America) in the framework of taxonomic revisions is needed to come to the appropriate taxonomic solution regarding Russian specimens of the genus.

Unlike *B. recurva*, *Symblepharis crispifolia* has rather compact East Asian distribution, mainly associated with coastal and insular areas of the Russian Far East, China, South Korea, and Japan. However, our results as well as splitstree topology published by Hedenäs [24] show that it is closely related to temperate North American species *S. raui*, which has distribution largely limited by the Appalachian region, the area considered to be the center of paleoendemism [39]. The morphological justification of this grouping includes saxicolous growth on acidic rocks, dark coloration, bistratose upper leaf lamina, and short setae. So, *S. crispifolia* + *S. raui* clades clearly demonstrate temperate East-Eastern disjunction, considered as characteristic for relicts of Arcto-Tertiary flora among bryophytes [32,40]. Within East Asian *S. crispifolia*, three distinct phylogenetic lineages were revealed; two of them are represented by both insular and inland populations, and one by specimens from Japan (two specimens studied by Hedenäs [24]). Morphological specimens from the Russian Far East differ from all other specimens, tentatively referred to as *S. crispifolia* s.str. in having shorter setae, mostly shorter than 4 mm but longer than 2 mm. However, according to [41], *Oncophorus crispifolius* var. *brevipes* (Cardot) Ihsiba has setae 1–2 mm long vs. 2–5 mm in *O. crispifolius* s.str., so Russian plants correspond to the latter, while Korean and Japanese specimens SyF14, SyF24, and SyF47 do not fit in the description, provided by [41], but agree with the description by [42]: “setae 4–5 (–10) mm long”. Moreover, in the clade, tentatively corresponding to var. *brevipes*, is where the specimen from Japan (SyF26) with fairly long setae, longer than 5 mm, landed. On the other hand, in having leaves, weaker widening proximally than in Japanese plants, several Russian specimens correspond to the protologue of *Dicranoweisia fauriei* Cardot [43] described from Korea (currently considered as a synonym of *S. crispifolia*), but Korean specimen SyF47 morphologically aligns with it even better, despite representing the same molecular lineage where two specimens of *S. crispifolia* s.str., SyF14 and SyF24, are found. Due to the high morphological variability, the name *S. crispifolia* has at least three synonyms recognized earlier at the species level [44]. However, due to the lack of fair diagnostic morphological characters delimiting the revealed molecular lineages, at the moment, we prefer to consider them as cryptic species. The degree of molecular divergence of lineages within *S. crispifolia* is lower than that among cryptic lineages of *Bellibarbula recurva* both in the cp *rps4*-*trnS* and nr ITS regions, so that the phylogenetic signal delimiting them largely originates from the highly polymorphic non-coding regions of cp DNA *trnF*-*trnT* and *trnG* and also from the intron in the mt Nad5 gene.

### 3.2. Are the Cryptic Species a Peculiar Trait of East Asian Moss Flora?

Since DNA sequencing became available, the number of studies dealing with cryptic speciation in many groups of living beings increased dramatically, indicating multiple cases of discordance between molecular data and morphology [45,46,47,48]. Among the main hypotheses of the cryptic species origin, recent divergence constrained morphological evolution under the limitation of the ecological niche and parallel/convergent evolution was proposed [45,47]. The impact of these mechanisms was assessed for several model groups such as amphipods, but it remains poorly understood in plants, where the cryptic diversity concept remains rarely applied [45]. At the same time, this mechanism was suspected to underly morphological similarity of vascular plants, demonstrating disjunctive distribution in East Asia and East North America [33,49].

Bryophytes were particularly mentioned by Bickford et al. [45] as a group, where the lumping approach led to under-recognizing species diversity that was partly corrected by a suite of recent integrative taxonomic studies [50,51,52,53,54,55,56], etc. Noteworthily, most of the revealed taxa were not cryptic, but appeared to have fair morphological circumscription. A few reported cases of true cryptic speciation mostly appeared in mosses from rich fens [57,58,59], or aquatic mosses [60]. However, already, the first morpho-molecular study of the broadly circumscribed temperate bryophyte taxa showing Madrean–Tethyan disjunctive distribution revealed incongruence between molecular and morphological data, neither of which supported ancient divergence, as constrained by the Madrean–Tethyan hypothesis. Therefore, recent dispersal events were proposed for all three studied species [61]; no morphological differentiation was found between European and West North American populations. In the other disjunct groups along with well morphologically separable groups of species, such as *Pulvigera*, *Orthotrichum tenellum*, and *Lewinskya affinis* [53,55,62], narrow species hardly distinguishable morphologically were reported [52].

At the same time, East Asian taxa were rarely involved in the integrative taxonomic studies with broad geographic sampling. Molecular phylogenetic studies by Huttunen et al. [63] and Ignatova et al. [64] showed that East Asian species of the genera *Homalothecium* (Brachytheciaceae) and *Isothecium* (Lembophyllaceae) are not related to European and North American ones; therefore, new genera were introduced to accommodate them. In flowering plants, disjunct East Asian–East North American pairs of species are rarely close relatives; instead, further diversification on one or both continents is found [33]. In bryophytes with such disjunction, a single study covered several accessions of the species with East Asian–East North American distribution, *Orthotrichum consobrinum*, and very weak divergence between two partitions of its range was found that was considered as evidence supporting long-distance dispersal [65]. A single North American accession of *Redfearnia homomallifolia* appeared to have nearly identical ITS2 sequence to one of the central Asian accessions of *R. baii* [66], but different ITS1. East Asian plants previously referred to the predominantly amphiatlantic *P. eugyrium* appeared to be close neither to European nor to East North American segregates of this species, while a single involved accession of *P. neglectum* was very close to the East Asian population of this species [9]. At the same time, a growing amount of evidence suggests the presence of unrecognized or incorrectly interpreted diversity of bryophytes in East Asia as exemplified by recent discoveries in the genera *Brachytheciastrum*, *Dicranella*, *Fissidens*, *Glyphomitrium*, *Weissia*, etc. [7,8,10,11,67]. Our results obtained for *Symblepharis crispifolia* and *S. raui* contribute to this trend and agree with the observation by [33], indicating further *S. crispifolia* divergence in East Asia. However, high morphological variability, which received only partial support from the molecular data; poor and controversial descriptions of taxa in the literature; and low accessibility of type material hinder the further taxonomic consideration of the revealed molecular segregates within *S. crispifolia* and also in several other East Asian taxa such as *Dicranella* cf. *heteromalla*, *Glyphomitrium*, and *Miyabea*. Strong molecular divergence between East and Central Asian populations of *Bellibarbula* and the isolated position of the East Asian (Russian) population indicate long-term isolation, which might justify the absence of *Bellibarbula* records between these partitions. However, weaker divergence between the East Asian and East North American population (as preliminarily assessed based on a single sequence available for the American population) seemingly deviates from this pattern and may reflect the impact of other mechanisms such as long-distance dispersal events, which is a matter for further study.

A lack of the morphological justification of the molecular divergence revealed in both studied groups might have been caused by the morphological stasis, which theoretically is more probable in temperate climate comparing with higher latitudes. East Asia is known as a region where humid forests occupy wholly the longitudinal interval from the tropics of Southeast Asia to the Arctic, supporting connections with tropic and boreal biomes [68]. One of the most diverse temperate flora of the world have developed here [69]; many endemics of the area represent evolutionary old taxa and are considered as Tertiary relicts [69,70]. On the other hand, the authors of [69] mention a bulk of studies, which revealed deep divergence between disjunct North Chinese plant populations, which was considered as a consequence of the long-term isolation in refugia. In the areas, spread northward, strong climatic gradients and dramatic changes in climate and landscapes during the late Tertiary and Quaternary have hardly favored speciation originated as a result of gradual divergence constrained by stabilizing selection [71], but rather led to new ecological niche formation and active peripatric speciation, which have enhanced morphological evolution. On the other hand, in the area, which experienced weaker environmental changes driven by climatic oscillations, they might have stimulated cryptic speciation due to fragmentation plant ranges.

## 4. Conclusions

We explored two mosses, found in the Russian Far East on the northern limit of their distribution and considered as relicts of Arcto-Tertiary flora due to possessing characteristic disjunction between East Asia and East North America. *Bellibarbula* cf. *recurva* is reported from Russia for the first time. Its population in the coastal area of the Sea of Japan is remarkably distinct from the closest Sino-Himalayan population, sampled early for molecular data. Sparse molecular data existing for the North American population indicate much higher similarity with our specimens. A lack of morphological differentiation limits further implication until denser sampling is made. We provide a bulk of new distribution data for *Symblepharis crispifolia*, previously known from the Russian Far East from a few old records. Despite morphological and molecular uniformity of Russian specimens, a high degree of morphological variation within this group is not congruent with the revealed phylogenetic structure. Therefore, three molecular lineages revealed within *S. crispifolia* are considered as cryptic. We suggest a high proportion of cryptic species in many bryophytes representing East Asian temperate flora due to the impact of morphological stasis, which is hypothesized to be higher as compared with higher latitudes.

## 5. Material and Methods

Initial datasets for molecular phylogenetic studies based on the results of a BLAST search (https://blast.ncbi.nlm.nih.gov/Blast.cgi?CMD=Web&PAGETYPE=BLASTHome (accessed on 10 November 2024)): After rough assessment of their affinities, three specimens presumably representing *Bellibarbula* were introduced in the dataset, used by [22], composed of nr ITS and cp *rps4*-*trnS* and *trnM*-*trnV* regions. Voucher data of the originally studied specimens and GenBank accession numbers for the newly generated sequences are provided in the “Specimens examined” paragraph. Three accessions of *Bellibarbula* from the Sino-Himalayan region, representing both species of the genus currently recognized by the Bryophyte Nomenclator project [72], allowed for estimating whether our specimens represent this genus. To assess phylogenetic affinities of the continental Asian specimens of *Symblepharis crispifolia*, we incorporated four originally studied accessions from the Primorsky Territory of Russia, tentatively referred to as “*O. crispifolius* var. *brevipes*” (SyF31, 54, 55, BF81), and four accessions possessing longer setae and therefore referred to as *S. crispifolia* s.str., two from South Korea (SyF14, SyF47), and two from Japan (SyF24, 26), in the dataset by [23], which included plastid *trnL*-*trnF* and *rps4* regions and an intron of the mitochondrial *Nad5* gene. The representation of taxa was limited to the R2 clade of the Rhabdoweisiaceae and added by the originally studied accessions of *Symblepharis sinensis* and *S. crispifolia* s.str. from East Asia. Additionally, cp *trnF*-*trnT*, *trnG*, and nuclear ITS sequences were obtained for the originally studied specimens with the support of Tsitsin Main Botanical Garden state assignment no. 122042500074-5, and several accessions studied by Hedenäs [24] were introduced based on sequences of *trnG*, *rps4*, and ITS, represented in GenBank, including two Japanese accessions of *S. crispifolia* (isolates P205, 206).

Voucher data of the originally studied specimens and GenBank accession numbers for the newly generated sequences are provided in Table 1. Both datasets were realigned manually using BioEdit [73] to account for changes in the representation of the involved lineages and to accommodate newly generated data. The newly obtained, for the Russian specimens of *Bellibarbula*, sequences of the ITS region showed a low degree of similarity with the closest sequences represented in GenBank and our attempts to introduce them in the alignment appeared to be problematic due to the lack of obvious homology between ITS sequences of *Bellibarbula* and those of other representatives of Pottiaceae. Also, in several places, reading of ITS was problematic due to paralogs. Therefore, the dataset was limited to two plastid markers. After excluding redundant accessions, the datasets included 78 terminals and 1435 positions for *Bellibarbula* and 32 terminals and 4088 positions for *Symblepharis.* Indel data were scored by using the simple indel coding (SIC) approach [74] in SeqState 1.4.1. [75] and added to the datasets prepared for Bayesian inferences. Partitioning and model settings followed [22] for the *Bellibarbula* dataset; the *Symblepharis* dataset was divided into three partitions, corresponding to the organellar data, nuclear data, and indels (used in Bayesian analysis). Bayesian analyses in MrBayes 3.2.7. [76] were set for 5 million generations and a sampling frequency of one tree each for 1000 generations; average standard deviations of split frequencies were checked to have decreased below 0.01 after the first 1 million generations. The chain temperature was set at 0.02 in all analyses, and the GTR model with sampling throughout the model space (setting nst = mixed) was used in all analyses. Convergence of the analyses was assessed via ESS values, checked using Tracer v.1.7.2. [77] to be higher than 200. Consensus trees were calculated after omitting the first 25% of trees as burn-in. ML trees were computed in iQ-tree [78] via the web server http://iqtree.cibiv.univie.ac.at/ (accessed on 20 November 2024) with 1000 generations of ultrafast bootstrapping and the same model of nucleotide substitutions (GTR + G + I) and partitioning approach, as was used for the Bayesian analysis.

## Figures and Tables

**Table 1:** GenBank accession numbers and IDs of specimens included in the phylogenetic analysis.

Species	Isolate	Country	Specimen_Voucher	*trnF*-*trnL*/*trnF*-*trnT*	*rps4*-*trnS*	*trnG*	*Nad5*	ITS
*Rhabdoweisia crispata*	RF3	Russia		MF353327	MN092565	-	MN092702	MF353249
*Rhabdoweisia crispata*	RF4	Russia		MF353328	MN092566	-	MN092703	MF353248
*Oreas martiana*	BF100	Russia		PQ139328	PQ139320	-	PQ139313	PQ144308
*Oreas martiana*	RF5	Russia		MN092431	MN092562	-	MN092698	MF353247
*Brideliella demetri*	DT414	Russia		OP485126	OP485145	-	-	OP495615
*Brideliella demetrii*	**BF84**	Russia: Taimyr, Anabar Plateau	**MW9050715**	**PQ593626**	PQ593592	**PQ593569**	**PQ593586**	-
*Brideliella wahlenbergii*	P237			-	LT576710	LT576606	-	LT576500
*Oncophorus virens*	DT415	Russia		MF353317	MN092554	-	MN092690	MF353238
*Oncophorus virens*	P575	Russia		-	MK456013	MK467015	-	MK456013
*Oncophorus integerrimus*	MSOv	Russia		MN092426	KX580552	-	KX580393	OP495626
*Oncophorus integerrimus*	On1561	Russia		MN092427	MN092553	-	MN092689	OP495627
*Symblepharis dendrophila*	P207	Russia		-	LT576680	LT576576	-	LT576471
*Symblepharis dendrophila*	P208	Russia		-	LT57668	LT576577	**-**	LT576472
*Symblepharis dendrophila*	On1923	Russia		MT756852	MT756859	-	MT756857	-
*Symblepharis raui*	**RF152**	USA: Kentucky	**Brinda 6319 (MW)**	MN718486	MN718547	**PQ593570**	MN718526	-
*Symblepharis raui*	P210	USA		-	LT576683	LT576579	-	LT576568
*Symblepharis raui*	P209	USA		-	LT576682	LT576578	-	LT576473
*Symblepharis crispifolia*	P206	Japan		-	LT576679	LT576575	-	LT576470
*Symblepharis crispifolia*	P205	Japan		-	LT576678	LT576574	-	LT576567
*Symblepharis crispifolia*	**SyF31**	Russia: Primorsky Territory	**Kedrovaya Pad’ Fedosov 29.IX.2023**	**PQ593627**	**PQ593593**	**PQ593571**	**PQ593587**	**PQ590644**
*Symblepharis crispifolia*	**SyF26**	Japan	**Bakalin Japan J-7-23-14 (VGBI)**	**PQ593628**	**PQ593594**	**PQ593572**	**PQ593588**	**PQ144311**
*Symblepharis crispifolia*	**BF81**	Russia: Primorsky Territory	**Ussurijsky Reserve, Fedosov *et al.*, 20.VIII.2022**	**PQ593629**	**PQ593595**	**PQ593573**	**PQ593589**	**PQ590645**
*Symblepharis crispifolia*	**SyF54**	Russia: Primorsky Territory	**Mramornaya Mt, Fedosov & Pisarenko, 21.IX.2024**	**PQ593630**	**PQ593596**	**PQ593574**	-	-
*Symblepharis crispifolia*	**SyF55**	Russia: Primorsky Territory	**Andreevka, Fedosov & Pisarenko, 18.IX.2024**	**PQ593631**	**PQ593597**	**PQ593575**	-	-
*Symblepharis crispifolia*	**SyF47**	South Korea	**24/0/3 S.S. Choi**	**PQ593632**	**PQ593598**	**PQ593576**	-	-
*Symblepharis crispifolia*	**SyF14**	South Korea	**Bakalin K-43-16-14 (VGBI)**	**PQ593633**	**PQ593599**	**PQ593577**	**PQ593590**	**PQ590642**
*Symblepharis crispifolia*	**SyF24**	Japan	**MW9050532**	**PQ593634**	**PQ593600**	**PQ593578**	**PQ593591**	**PQ590643**
*Symblepharis vaginata*	**RF150**	USA: New Mexico	**Brinda 13,613 (MW)**	MN718484	MN718545	**PQ593579**	MN718524	OL435100
*Symblepharis vaginata*	**RF151**	USA: Arizona	**Brinda 13,443 (MW)**	MN718485	MN718546	**PQ593580**	MN718525	OP495629
*Symblepharis vaginata*	**SyF30**	Russia: Buryatia Republic	**Mondy settl. Fedosov & Pisarenko, 11.VII.2023**	**PQ593635**	**PQ593601**	**PQ593582**	-	**PQ590647**
*Symblepharis sinensis*	**SyF17**	Russia: Buryatia Republic	**Mondy settl. Fedosov & Pisarenko, 11.VII.2023**	**PQ593636**	**PQ593602**	**PQ593583**	-	**PQ590646**
*Symblepharis sinensis*	**RF25**	Russia: Dagestan Republic	**MW9050696**	MF353319	MN092550	**PQ593584**	MN092686	MF353240

Originally studied specimens are boldfaced and annotated with provenance and voucher information.

## Data Availability

Data are contained within the article.
